# The Use of Functional Genomics in Conjunction with Metabolomics for *Mycobacterium tuberculosis* Research

**DOI:** 10.1155/2014/124218

**Published:** 2014-03-18

**Authors:** Conrad C. Swanepoel, Du Toit Loots

**Affiliations:** School for Physical and Chemical Sciences, Centre for Human Metabonomics, North-West University, Private Bag x6001, Box 269, Potchefstroom 2531, South Africa

## Abstract

Tuberculosis (TB), caused by *Mycobacterium tuberculosis*, is a fatal infectious disease, resulting in 1.4 million deaths globally per annum. Over the past three decades, genomic studies have been conducted in an attempt to elucidate the functionality of the genome of the pathogen. However, many aspects of this complex genome remain largely unexplored, as approaches like genomics, proteomics, and transcriptomics have failed to characterize them successfully. In turn, metabolomics, which is relatively new to the “omics” revolution, has shown great potential for investigating biological systems or their modifications. Furthermore, when these data are interpreted in combination with previously acquired genomics, proteomics and transcriptomics data, using what is termed a systems biology approach, a more holistic understanding of these systems can be achieved. In this review we discuss how metabolomics has contributed so far to characterizing TB, with emphasis on the resulting improved elucidation of *M. tuberculosis* in terms of (1) metabolism, (2) growth and replication, (3) pathogenicity, and (4) drug resistance, from the perspective of systems biology.

## 1. Introduction

Tuberculosis (TB), caused by* Mycobacterium tuberculosis*, is considered to be the world's second most deadly infectious disease, after that for which the human immunodeficiency virus (HIV) is responsible. The pathogen was discovered in 1882 by the German physician Robert Koch [1, 2]. The World Health Organization (WHO) recently reported that one-third of the global population is infected with* M. tuberculosis* [2] and in 2010 documented 8.8 million new cases of TB, 650 000 of which were infected with multidrug-resistant (MDR) strains, contributing to the 1.4 million deaths reported globally (equivalent to 3 800 deaths a day) for that year. Developing countries are by far the worst affected by this epidemic and account for 95% of the reported global deaths due to this disease [3]. Additionally, 25% of the individuals who succumb as a result of this epidemic are reported to be coinfected with HIV, with Europe and Africa exhibiting the highest HIV coinfection rate [4]. Although the mortality rates of these coinfected individuals have declined since the advent of antiretroviral therapy, MDR-TB still contributes greatly to the high mortality rates in the rural areas of especially southern Africa, as a direct consequence of these patients failing to comply with the treatment regimens [5, 6]. These findings are alarming, as TB is considered a curable disease and those affected are capable of a full recovery [7, 8] after the successful completion of the correct treatment regimens [9].

By applying different research methodologies (such as genomics, proteomics, transcriptomics, and lipidomics), a large body of knowledge has been generated and applied to new diagnostic and treatment protocols for infectious diseases, including TB [10, 11]. The latest addition to these “omics” methodologies, metabolomics, can be defined as the unbiased identification and quantification of all metabolites present in a biological sample (the metabolome) at a given time, using highly sensitive and selective analytical techniques [12], followed by the interpretation and visualization of the data generated via biostatistics [13]. Metabolic profiling, defined as the “detailed metabolome analysis requiring highly specialized analytical techniques and accurate concentration determination for sample classification” [14], and metabonomics, “the quantitative measurement of the dynamic multiparametric metabolic response of living systems to pathophysiological stimuli or genetic modification” [15], are key terms used to expand the definition of metabolomics [12]. Alterations in the metabolic profile of an organism can be directly linked to the corresponding genes in its genome, as Raamsdonk et al. [16] illustrated with the Functional Analysis by Coresponses in Yeast (FANCY) approach. Their method proves the principle that any genetic modification of an organism's functional gene will lead to alterations in its metabolite profiles [17]. Moreover, the FANCY approach provides additional information towards elucidating gene function by comparing the metabolic profiles of different organism strains with known or unknown gene modifications and processing the metabolite data generated using multivariate statistical analyses, in particular principal component analysis (PCA) and partial least squares discriminant analysis (PLS-DA), in order to identify those metabolite markers best accounting for the differences between strains [17, 18]. When used in conjunction with enzymology and proteomics, this approach can assist in deciphering enzyme/protein functionality by comparing the metabolic profiles of a wild-type strain to those of an identical strain with a deletion in the genome corresponding to a gene coding for the specific enzyme/protein involved [19]. The advantages of using metabolomics for such comparative investigations are that (1) it provides an excellent representation of the cellular metabolite state of an organism at the time of sampling, and of the influence of any perturbation induced by the environment, altered genes, or disease [20, 21]; (2) metabolites can be identified with a high degree of certainty and there are fewer metabolite types/classes that exist than there are genes or proteins [22]; and (3) thanks to the excellent analytical techniques currently available, accurate characterization and quantification of the metabolome can be achieved, using only small sample amounts, with minimum sample preparation beforehand [23, 24]. The most commonly used analytical techniques for metabolome/metabolic profile analyses are nuclear magnetic resonance (NMR) and mass spectrometry (MS) [23]. NMR provides a unique advantage in that it is nondestructive of the sample being analyzed and is the preferred technique when characterizing unknown compounds [25]. NMR spectra, however, are considered rather complex; when investigating metabolic profiles containing a great diversity of metabolites with many compound classes and concentrations [26], these criteria are judged to be less useful. For these applications, MS is the preferred approach, as it is less time consuming and simplifies detection. A further advantage of using MS for such applications is that it can be used to elucidate and monitor the metabolic pathway of a substrate and its intermediates active in metabolism, by labeling them with stable ^13^C isotopes.

The addition of chromatographic separation of the compounds in a complex sample, prior to MS detection [27], greatly improves the sensitivity and accuracy of such analyses. Various “hyphenated” MS approaches exist for such applications and include those combined with gas and liquid chromatography, enabling the separation of the compounds in a mixture on the basis of such chemical properties as their solubility, volatility, and mass [23, 24]. Deconvolution of overlapping spectral peaks, in combination with accurate peak identification, further improves metabolome analyses and subsequently contributes to the better elucidation of the metabolic profiles [24].

Interpretation of the metabolomics data, in combination with those generated using other “omics” approaches, collectively defined as a systems biology research approach, provides a holistic view of the organism or cell under investigation [20, 28]. All the information generated from such research disciplines is used in combination to explain the phenomenon or perturbation of interest. In this review we discuss the previously reported functional genomics research conducted, in conjunction with recently generated metabolomics data, in an attempt to improve the characterization of* M. tuberculosis*. A detailed overview of the gene encoding entities contributing to (1) metabolism, (2) growth and replication, (3) pathogenicity, and (4) drug resistance of this microbe will be considered relevant. We then highlight those areas not yet fully understood and the potential role metabolomics can play in elucidating these.

## 2. Interpreting Functional Genomics in Conjunction with Metabolomics for Improved Characterization of* Mycobacterium tuberculosis*

The* M. tuberculosis* complex can be separated into a variety of subspecies, namely,* M. tuberculosis, M. canetti, M. africanum*,* M. microti, M. caprae, M pinnipedii, M. bovis,* and* M. bovis* Bacille Calmette Guerin (BCG) [29], all of which can potentially cause a TB-like disease state in mammals. Interestingly, these subspecies represent 0.01% genetic diversity in terms of their respective genomes [30]. Factors contributing to the minor genetic variation observed between these mycobacterial species include (1) differences in the copy number and location of an insertion sequence (IS) specific for* M. tuberculosis *and* M. bovis* (IS6*110*); (2) variations in a subgroup of the Pro-Glu (PE) family of proteins, namely, polymorphic GC-rich sequences (PGRS); and (3) the variation observed in short DNA sequences, characterized as the region of difference (RD) [31, 32]. The genome of* M. tuberculosis* exhibits more than 4 million nucleotide base pairs (bp), with guanine and cytosine the major components (65.6%), in addition to over 4 000 genes [33], 82 of which were added following the reannotation of the genome by Camus [34].

Considering these differences, one of the first TB metabolomics investigations conducted by the Laboratory of Infectious Disease Metabolomics, Centre for Human Metabonomics, North-West University, South Africa, was an initial test of the functionality of this approach for detecting minor variations in the metabolomes of different* Mycobacterium* species, including* M. tuberculosis*,* M. kansasii*,* M. avium*, and* M. bovis* BCG, on the basis of the small genetic differences previously described between these species. Olivier and Loots [35] subsequently extracted the lipidome of these species and after successfully processing the data generated by gas chromatography-mass spectrometry (GC-MS), determined that these species could be differentiated from one another on the basis of their characteristic metabolite signatures, using a combination of 12 selected metabolite markers. More importantly, these markers were subsequently used to construct a diagnostic model able to identify these* Mycobacterium* species in sputum collected from patients, further highlighting the potential of metabolomics for diagnosis. These results not only showed that the minor genetic variations observed result in a significantly altered metabolite profile but also that metabolomics is sensitive enough to detect these changes. Later studies using this approach included metabolomics investigations of the functionality changes associated with virulence (ESX-1) and growth (ESX-3) [36], factors contributing to* M. tuberculosis* drug resistance [37], hyper- versus hypo-virulence [38], and adaptations of the host to* M. tuberculosis* and vice versa [39]. These topics are discussed in greater detail below in addition to some of the significant contributions to TB metabolomics by other research groups.

### 2.1. Systems Biology Related to General* M. tuberculosis* Metabolism

#### 2.1.1. The Tricarboxylic Acid, Glyoxylate, and Citramalic Acid Cycles

Although some metabolic pathways in* M. tuberculosis* differ from those of other bacterial species, the microbe's genome has all the elements and pathways necessary to synthesize essential biomolecules (amino acids, vitamins, and enzyme cofactors) necessary for replication and growth [30, 33]. Evidence from various genomic studies showed that the H37Rv strain of* M. tuberculosis* is capable of metabolizing carbohydrates, hydrocarbons, alcohols, ketones, and carboxylic acids [33] via the glycolysis pathway, the pentose phosphate pathway, the tricarboxylic acid (TCA) cycle, and the glyoxylate cycle, respectively [33, 40]. Subsequently, Tian et al. [41] identified a new TCA cycle in* M. tuberculosis* using a biomedical metabolomics approach [42]. In their study they indicated that* M. tuberculosis *shows no *α*-ketogluturate dehydrogenase (KDH) activity and subsequently proved the existence of a split TCA cycle, with or without interconnecting pathways. This split originates after the decarboxylation of *α*-ketogluturate to the conventional TCA intermediate, succinate, via (1) succinic semialdehyde (SSA) or (2) glutamate's conversion to 4-aminobutyrate (GABA) and then SSA [41].

As mentioned above, ^13^C-labeled metabolites/substrates permit the metabolic pathway to be elucidated and the metabolism of a substrate and its intermediates to be monitored [19, 43]. When used in combination with different genomic techniques, this approach may serve to clarify better the functionality of uncharacterized genes. Such a multifaceted approach was used successfully to determine the functionality of Rv1248c (*sucA*), which was originally believed to be the E1 component of the KDH complex. Prior to this, various genomic studies reported a lack of the E2 component of KDH, namely, dihydrolipoamide succinyltransferase. This compound was thought to render this enzyme complex inactive in* M. tuberculosis* and was subsequently proposed as an alternative working model for Rv1248c, which was believed to be responsible for the nonoxidative decarboxylation of *α*-ketogluturate to SSA. The enzymatic conversion of substrate to product by the recombinant Rv1248c was monitored using ^1^H-NMR combined with activity-based metabolite profiling [43, 44]. Subsequently, Rv1248c was shown to be a carboligase enzyme, responsible for catalyzing the synthesis of 2-hydroxy-3-oxoadipate (HOA), by condensing the activated aldehyde group of *α*-ketoglutarate with glyoxylate [43, 45]. Of even greater importance was the realization that this approach could be used to elucidate the functionalities of other enzymes and their associated genes. Subsequently, the functionality of phosphoenol-pyruvate carboxykinase (PEPCK), encoded by* pckA* [46, 47], which was previously believed to be responsible for catalyzing the interconversion of oxaloacetate (OAA) and phosphoenol-pyruvate (PEP), was determined. Examining* M. bovis *BCG, using metabolite profiling of ^13^C-labeled acetate carbon flux in wild-type and the* pckA*-deleted strains, revealed that this enzyme is responsible for only the unidirectional conversion of OAA to PEP [47]. This proved that although the TCA cycle produced pyruvate, it was not converted to PEP [43] as was previously thought. These findings further illustrated a lack of pyruvate phosphate dikinase (*ppdK*) activity in these organisms [47] and led to the conclusion that PEPCK was involved in gluconeogenesis in* M. tuberculosis *[48]. Further application of this “omics” approach revealed that during* in vitro *growth of* M. tuberculosis*, carbon flow between known intermediates of the TCA cycle, *α*-ketoglutarate, and succinate was intermittent, further confirming the lack of a functional KDH enzyme and that this organism operates via a bifurcated TCA cycle [43, 49]. Olszewski et al. [50] subsequently illustrated the same phenomenon in* Plasmodium falciparum*, the causative agent of malaria [51], using a similar metabolomics approach. They showed that this protozoan parasite also has a bifurcated TCA cycle at *α*-ketoglutarate, with the resultant two pathways simplifying the degradation of glutamate and/or glutamine into two carbon units [43].

Whereas the early genomics methods identified various genes related to the TCA cycle, their exact functionality was only truly determined after metabolic examinations, confirming how crucial metabolomics and the interpretation of these data using a systems biology approach can be.

#### 2.1.2. Lipid Metabolism

*Mycobacterium tuberculosis* possesses a great number of lipophilic molecules, ranging from common fatty acids to glycolipids [52] and very-long-chain molecules (such as mycolic acids) [33, 53]. Although many enzymes have been identified as being directly responsible for the biosynthesis of these lipids, they are greatly outnumbered by those responsible for fatty acid oxidation in these organisms. Previous genomics studies revealed that the genome of* M. tuberculosis* encodes more than 250 enzymes related to fatty acid oxidation, which is remarkable considering that the corresponding number for* E. coli* is only 50 [33]. In* M. tuberculosis*, lipids are generally metabolized to yield energy essential for mycobacterial growth and replication. Many, however, are also used in the synthesis of various components directly involved with the mycobacterial cell wall, which in turn plays a particular role in mycobacterial virulence, as will be explained in greater detail in what follows. The majority of complexes and enzymes required for lipid degradation, are encoded for by a vast amount of these genes and include (1) 36 acyl-CoA synthases; (2) 36 acyl-CoA dehydrogenases (capable of catalyzing the initial step in fatty acid degradation); (3) 21 enzymes associated with the enoyl-CoA hydratase/isomerase superfamily; (4) six 3-hydroxyacyl-CoA dehydrogenases [54] (responsible for the conversion of 3-hydroxy fatty acids to 3-keto-fatty acids) [33, 55]; and (5) six acetyl-CoA C-acetyltransferases, completing *β*-oxidation, eventually to yield two acetyl-CoA molecules [54].

Complementary to these findings is the more recently acquired metabolite information associated with these genes. More specifically, various predictions regarding the associated carboxylase systems have been made from this perspective but once again with considerable uncertainty [34]. Pyruvate carboxylase (PYC), encoded for by* pycA*, is thought to be necessary for the interconversion of oxaloacetate and pyruvate. The latter in turn can be metabolized to yield acetyl-CoA, the same end product following the catabolism of fatty acids via *β*-oxidation [45, 56]. Acyl-CoA carboxylase (ACC), on the other hand, has been shown to be responsible for the carboxylation of more specific short-chain acyl-CoA substrates to their respective lipid products, subsequently serving as substrates in the fatty acid synthase (FAS) systems and polyketide synthesis, ultimately producing mycolic acids [57]. Interestingly, both PYC and ACC require biotin as cofactor, with this vitamin donating a carboxyl anion to the very specific organic acid substrate [45, 57]. There is limited information on PYC in the metabolomics literature, but ACC has been the subject of more metabolic research due to it potentially serving as an antibiotic drug target, which Rabinowitz and coworkers [58] illustrated using isotope tracer metabolites and a metabolomics research approach.

Additional metabolomics-related approaches by de Carvalho et al. [19] pertaining to these genes associated with fatty acids revealed compartmentalized cocatabolism of carbon substrates in* M. tuberculosis* and a description of how various carbohydrates and fatty acids can be channeled simultaneously to their respective metabolic fates. They subsequently provided an entirely new understanding of this previously uncharacterized metabolic network. Using ^13^C labeling of the metabolites, these authors confirmed that* M. tuberculosis* is not capable of diauxic growth and that the pathogen has the capacity to catabolize multiple carbon sources simultaneously, ensuring optimal monophasic growth. de Carvalho et al. [19] further concluded that each individual isotopically labeled carbon source, added to the growth media of the* M. tuberculosis* H37Rv cells, was successfully and differentially catabolized through the glycolytic, pentose phosphate or TCA pathways.

Further genomic studies illustrated two related enzyme systems as responsible for synthesizing essential fatty acids in mycobacteria, namely, (1) fatty acid synthase I (FASI) [59] and (2) fatty acid synthase II (FASII) [33, 60]. In contrast to the mycobacterial FASII system, FASI exhibits a multidomain enzyme necessary for fatty acid biosynthesis [61]. Interestingly, mycobacteria are the first prokaryote organisms in which both of these systems were found to be fully functional [61]. Genes encoding acyl carrier proteins (ACPs) were also identified in the genome and these proteins were shown to function by transporting all pathway intermediates between the active site centers of enzymes involved in the FASII biosynthetic system [59, 61]. The two systems function in unison: FASI synthesizes precursors necessary for mycolic acids and other complex lipids present in the cell wall [33, 62]; and FASII elongates the FASI products to form meromycolate precursors, which are chemically altered eventually to form mycolic acids [61–63]. As part of this elongation process, *β*-ketoacyl-ACP synthase (*KasA*) catalyzes the condensation of the two carbon units from malonyl-ACP to a rapidly growing chain characterized as acyl-ACPs [64]. Interestingly, in contrast to the metabolism of mycobacteria, the host FASII differs from FASI in that the latter uses a single multifunctional enzyme-ACP complex to initiate fatty acid biosynthesis [65]. Moreover, the characteristic differences between the structural arrangement of bacterial and mammalian synthases provide an attractive and unique opportunity for the development of antibacterial agents specifically targeting the bacterial FASII system. In addition, as these lipids make up approximately 60% of the mycobacterial cell wall [55], they are thought to play an important role in the viability and virulence of the organism, making them attractive targets in TB drug discovery [66].

Recently, du Preez and Loots [37] used a metabolomics approach to compare the lipid metabolomes of two genetically different* rpoB* mutant* M. tuberculosis* strains (S552L and S531L) to that of an isogenic* M. tuberculosis* wild-type strain, in an effort to characterize rifampicin resistance more fully. These authors subsequently detected reduced concentrations of various 10-methyl branched-chain fatty acids and the corresponding cell wall lipids in the metabolome of the mutant strains [37]. Their results suggested an inhibition of FASII and S-adenosylmethionine (SAM), which was confirmed by detection of elevated substrate concentrations in these enzyme pathways. Additionally, a decrease in various straight-chain fatty acids implied their increased utilization as an alternative energy source during the reduced growth rates associated with these mutants. The findings of this metabolomics investigation confirmed other studies indicating that the resistance of* M. tuberculosis* to rifampicin, due to a mutation in the *β*-subunit of the* rpoB* gene of RNA polymerase, comes at an overall fitness cost. These mutant organisms show reduced growth, replication, and viability, accompanied by various metabolic modifications in order to survive this state. This study was the first of its kind to indicate that the* M. tuberculosis rpoB* mutant strain is accompanied by an altered fatty acid metabolism and further illustrated the potential of metabolomics as a valuable genomics tool, able to elucidate gene functionality. These findings will be discussed further under systems biology related to drug resistance.

Lastly, genes responsible for encoding 9 polyketide synthase enzymes (*pks*) were also identified in the* M. tuberculosis* genome [60]. Polyketides are multidomain proteins essential for the construction of complex lipids and various metabolites associated with the mycobacterial cell envelope [67, 68]. Interestingly, the* pks *gene complexis located directly upstream of* mas*, and both their respective products—phthiocerol and mycocerosic acid—are used to produce another necessary cell wall component, phthiocerol dimycocerosate (PDIM), which is also strongly associated with mycobacterial virulence [33, 52]. Bisson and associates [69] illustrated a significant upregulation of the polyketide synthase genes,* ppsA-ppsE, *and* drrA *(Rv2936), responsible for the transport of PDIM over the mycobacterial cell envelope in Beijing and Haarlem* rpoB *mutant* M. tuberculosis* strains, using a combined metabolomics and proteomics approach. Although intact PDIM was not detected in either of the* rpoB* mutant strains used by Bisson and colleagues [69], elevated concentrations of the various precursors to this were recorded [67]. It was further hypothesized by Cole et al. [33] that these enzymes elongate FASI-CoA primers to produce the associated fatty acids. However, this could not be confirmed as the related metabolite data are inadequate for the purpose [33].

### 2.2. Systems Biology Related to Growth, Replication, and Survival

Five copies of the ESX gene cluster (ESX-1–ESX-5), collectively termed the ESAT-6 gene cluster region, have been identified in the genome of* M. tuberculosis* [70]. It is believed that six genes located within the cluster encoding the essential components of Type VII secretion (T7S) system are responsible for the transport of virulence and growth associated proteins across the highly hydrophobic cell wall of the organism [68, 71]. These five copies function independently from one another. Whereas ESX-1 and ESX-5 have been linked to virulence of* M. tuberculosis*, ESX-3 has been implicated in the uptake of iron and zinc by the pathogen [36, 72, 73]. The nine genes associated with the latter, Rv0282–Rv0292 [36], are under the transcriptional regulation of IdeR (iron-dependent repressor) and Zur (zinc-uptake regulator). Their subsequent levels of regulation are determined by the immediate concentrations of zinc and iron, respectively [74]. The exact role of ESX-3 in the T7S system, as well as its role in divalent cation homeostasis and growth, however, remains unclear. With regard to the different ESX gene clusters, ESX-3 is considered the only region essential for the* in vitro* growth of* M. tuberculosis *[75]. In addition, intracellular iron acts as a cofactor for over 30 enzymes in this pathogen and together with zinc fulfills critical roles in the activation of enzymes involved in amino acid biosynthesis in particular [76]. Subsequent to these findings, Loots and coworkers [36] used a metabolomics approach to explore the functionality of the ESX-3 gene cluster, using the nonpathogenic* M. smegmatis *strain as a model. This was achieved by comparing the metabolite profiles of an ESX-3 knock-out with those of a wild-type parent strain. Loots and colleagues [36] anticipated both iron and zinc starvation in the ESX-3 knock-out strain, due to their previously described role in the homeostasis of these essential divalent ions. This was the case for zinc, with the metabolite products synthesized using zinc-dependent enzymes detected in greatly reduced concentrations in the ESX-3 knock-out strain. Interestingly, iron illustrated the completely opposite behavior. This was ascribed to upregulation of other iron acquisition genes of* M. smegmatis,* overcompensating for the loss of ESX-3. This study of Loots et al. [36] successfully combined metabolomics and functional genomics and led to a better understanding of the role of iron and zinc in the growth of* M. smegmatis *and the functionality of their associated genes.

Various environmental and metabolic challenges threaten the viability of* M. tuberculosis.* This organism has accordingly developed an extensive array of regulatory factors to adapt and survive [33]. Following the discovery of 3 new genes in 2002 [34], the genome is now known to exhibit 11 pairs of genes related to the sensor histidine kinases and their associated response regulators [33]. The regulatory proteins present in the genome of the H37Rv strain are now known to exceed 100, with 13 sigma factors being responsible for supervising gene expression at the level of transcription initiation [33]. These transmembrane proteins have been linked to virulence in some fungal strains identified in immunocompromised individuals [77]. Also identified in the genome of* M. tuberculosis* are genes named* pknA*–*pknL*, thought to be responsible for coding 11 serine/threonine kinases (STPKs) [78, 79], known to act as sensors to environmental signals that thereby regulate developmental changes and host*-*pathogen interactions [80]. Further discovery revealed* pknA* and* pknB* to be upregulated during mycobacterial growth, and both form part of an operon, encoding genes necessary for the control of cell shape and ultimately cell wall synthesis [78]. Interestingly, within the* Mycobacterium* genus, the number of these kinase enzymes varies between 4 and 24, depending on the species [80], with* M. smegmatis* exhibiting no homologue proteins encoded for by the* pknF* and* pknG* genes [81]. Furthermore, these enzymes and the regulatory proteins mentioned above are involved in regulating different stress responses and control mycobacterial growth and pathogenicity, as will be discussed below.

Although not directly working on these* pkn* genes specifically, du Preez and Loots [37] also investigated bacteria-host interactions and adaptions using a metabolomics research approach. They reported for the first time the existence of a citramalic acid cycle in* M. tuberculosis*. They speculated, on the basis of this cycle's function in* Rhodospirillum rubrum*,that* M. tuberculosis* uses this as an additional means for generating carbon intermediates for energy production in the TCA cycle, as a means of surviving attack by the host. du Preez and Loots [37] also detected elevated concentrations of GABA and subsequently the importance of the glutamine - GABA shunt as a major source of carbon intermediates in the TCA cycle via succinic acid. Additionally, their metabolomics investigation detected adaptions of the host to the pathogen, which included a more than 10-fold elevation of various neurotransmitters, including norepinephrine, and postulated this as an additional mechanism for hydrogen peroxide synthesis via glucose autooxidation, as a means of eliminating the bacteria [37].

### 2.3. Systems Biology Related to Mycobacterial Pathogenicity

Also identified in the genome of* M. tuberculosis* are many genes coding for proteins strongly associated with virulence and pathogenicity. The identification of these genes stimulated further research regarding their respective products and functioning and considered their influence on both metabolism and their role in pathogenicity. Cole et al. [33] deduced that approximately 7.1% of the total coding capacity of the* M. tuberculosis* genome is devoted to the 169 PE and Pro-Pro-Glu (PPE) proteins, which are unique to mycobacteria. Their functions and location in the cell, however, are unknown [82]. Their names were derived from the Pro-Glu and Pro-Pro-Glu motifs identified near the N-terminus of these macromolecules. Due to their variation, the PE family of proteins are assigned to three groups, with most of the proteins belonging to the subclass PGRS [33, 83], whereas the majority of the PPE proteins exhibit major polymorphic tandem repeats (MPTR) [33, 82, 83]. Although not yet confirmed, it has been widely postulated that members of this extensive family are associated with immune evasion, antigenic variation, and virulence [84]. Additionally, some are thought to function as immunodominant proteins [82] or have been linked with the mycobacterial cell wall, as necessary entities for lipolysis and lipogenesis of host macrophages [33] following infection, subsequently promoting replication of the tubercle bacilli. Furthermore, various members of the Rv2123 family have been identified as being upregulated during conditions of depleted iron [82], whereas others were confirmed to play a role in disease pathogenesis by the upregulation of PPE41 [85]. Furthermore, other PE (Rv3872/PE35) and PPE (Rv1807/PPE31 and Rv3873/PPE68) proteins are thought to be necessary for the* in vivo* growth of* M. tuberculosis,* following infection of the host [82]. A further 3 PE (Rv0285/PE5, Rv0335c/PE6, and Rv1169c/PE11) and 5 PPE (Rv0286/PPE4, Rv0755c/PPE12, Rv1753c/PPE2, Rv3135/PPE50, and Rv3343c/PPE54) proteins were identified to be essential for the* in vitro* growth of the organism [75]. Interestingly, Rv3872 (PE35) and Rv3873 (PPE68) are encoded by the ESX-1 gene cluster region, strongly linked to the microbe's virulence and pathogenicity [82, 83]. As most of the PE and PPE proteins are encoded for by ESX-5, and these proteins are also related to pathogenicity, the result was not unexpected because no ESX-5 region was found to be present in the genome of nonpathogenic* Mycobacterium *species (such as* M. smegmatis* and* M. vanbaalenii*) [82]. Despite this, however, the fact that the virulent* M. bovis* strain exhibits a fully functional ESX-1 and ESX-5 gene cluster, gene alterations within the ESX-1 gene cluster region of this organism led to the construction of the* M. bovis* BCG vaccine strain, confirming that both these two gene clusters are required for pathogenicity and virulence [86]. Further research regarding the functionality, location, and eventual secretion of all PE and PPE proteins is therefore highly desirable, as these proteins are undoubtedly important to pathogenicity. Elucidating their exact function is considered essential for developing better vaccination and treatment strategies in the fight against the growing TB epidemic.

In the light of this, Meissner-Roloff and associates [38] compared the metabolomes of a hypo- and hypervirulent Beijing* M. tuberculosis* strain and subsequently identified a reduction in various metabolite markers in the relatively hypervirulent strain. Glutamine, alanine, and glycine, all precursors of glutamic acid, are characterized as the fundamental building blocks of the PGRS subfamily. The reduced concentration of these amino acids detected in the hypervirulent strain was ascribed to an increase in PGRS formation and hence to the associated virulence. Further confirmation for this was the reduced concentrations of alanine and glycine in the same strain, due to their role as a dipeptide (glycylalanine) in PGRS protein formation. These results not only confirmed the postulated role of PE and PPE proteins in virulence and the altered metabolism associated with them, but the researchers also identified a further 33 metabolite markers and thereby explained how an altered metabolic rate, varying metabolism related to growth and replication, cell wall synthesis, and antioxidant capacity, relates to the increased virulence associated with these strains [38]. Briefly, the reductions in various primary energy substrates—such as glucose, galactose, mannose, mannonic acid, myo-inositol, glutamine and glycerol—in the hypervirulent strain suggests an increased utilization of these compounds due to an elevated metabolic and growth rate in this organism in addition to the increased use of these and other structural metabolites—such as glycerol, galactofuranoside, arabinofuranose, cadaverine, alanine-glutamic acid, aspartic acid, and lysine—for cell wall synthesis [87, 88]. Reduced concentrations of commonly occurring fatty acids in the hypervirulent strain were ascribed to the previously reported upregulation of transcription factor Rv3574 and the dormancy survival regulon (DosR) [89], both of which are known to be involved in lipid metabolism and respiration. Lastly, reductions of phenylalanine and tyrosine, intermediates in the shikimate pathway and responsible for the synthesis of a number of compounds associated with virulence, in addition to alterations in the precursors of the antioxidant mycothiol (myo-inositol, glucoseamine, and the cysteine precursor, serine), indicated increased utilization of these in the synthesis of various factors associated with virulence [90].

### 2.4. Systems Biology Related to Drug Resistance

The first-line anti-TB drugs, isoniazid, ethambutol, pyrazinamide, streptomycin, and rifampicin [91, 92], target different processes in all mycobacteria that cause TB. These metabolic processes targeted by the above mentioned drugs are considered essential in mycobacteria for viability, pathogenesis, cell wall synthesis, protein synthesis, DNA replication, and extracellular transport [93]. The biosynthesis of fatty acids is another pathway essential for bacterial survival and includes the previously described FASII pathway, which is also considered an important target for anti-TB drug discovery [65]. Isoniazid and ethambutol have been shown to inhibit the cell wall synthesis of* M. tuberculosis*—isoniazid inhibits the synthesis of mycolic acids [9, 92] and ethambutol inhibits the synthesis of aribinogalactan in the cell wall [92], both of which are essential components of the mycomembrane [55, 94] and necessary for secreting various virulence-associated proteins via the T7S system [71]. Pyrazinamide, a structural analogue of nicotinamide, targets energy metabolism, also via disruption of the extracellular membrane of* M. tuberculosis* [9, 92]. Lastly, streptomycin and rifampicin both inhibit the synthesis of proteins and nucleic acids [9]; streptomycin interferes more specifically with protein biosynthesis, inhibiting the initiation of mRNA translation [92], whereas rifampicin interferes with bacterial RNA synthesis, effectively inhibiting the transcription of bacterial DNA to RNA by binding to DNA-dependent RNA polymerase**β**-subunit encoded for by the* rpoB *gene [9, 95].

Mutations in the* M. tuberculosis *genome, or any other organism capable of causing TB-like disease states in mammals, may potentially lead to resistance to these first-line antibiotics. Mutations in the gene encoding enoyl-acyl carrier protein reductase (*inhA*), an essential enzyme in FASII, are thought to lead to the subsequent resistance of* M. tuberculosis* to isoniazid [61]. Furthermore, Zhang et al. [96] indicated that a Ser315Thr mutation in the primary mycobacterial catalase-peroxidase gene,* katG,* also results in resistance, which was confirmed by drastically lowered catalase and peroxidase activities in the strains exhibiting this mutation [97]. Resistance to ethambutol, on the other hand, is thought to be due to a mutation at amino acid residue 306 of the* EmbB* gene (Met306Leu, Met306Val, and Met306Ile), responsible for the synthesis of arabinogalactan [98]. The majority (72–97%) of the pyrazinamide-resistant strains are characterized by a mutation in the gene coding for pyrazinamidase (*pncA*), responsible for the conversion of pyrazinamide to its active form, pyrazinoic acid (POA) [97, 99]. Other defective mechanisms responsible for metabolizing pyrazinamide and regulating the* pncA* gene and/or the efflux or POA have also contributed to the high level of pyrazinamide resistance observed in some clinical isolates, confirming that pyrazinamide resistance in* M. tuberculosis* is not due only to a single factor [100]. Streptomycin resistance, however, has been shown to result from one or more single point mutations: 491 (C→T), 512 (C→T) and 904 (A→G) in the 16S rRNA gene (*rrs*) coding for the ribosomal protein S12 (*rpsL*) [9] in addition to a Lys88Gln mutation that illustrates the same phenotypic effect. Infection with* M. tuberculosis *resistant to rifampicin is usually accompanied by higher fatality rates compared to other first-line medications [97], as resistance to rifampicin is usually accompanied by resistance to one or more of the other first-line antibiotics as well [95]. Mutations in the *β*-subunit of the RNA polymerase encoding gene* rpoB*, an enzyme crucial for the transcription of bacterial DNA to RNA, have been shown to cause rifampicin resistance [37, 95]. Furthermore, a variety of additional gene mutations in the 81-bp region of the* rpoB* gene, known as the rifampicin-resistance determining region (RRDR), have also been identified as resulting in the same phenotype [101].

Only two studies have investigated drug resistance in organisms responsible for TB from a metabolomics perspective. du Preez and Loots [37] researched the altered metabolic profiles of two different* rpoB* mutant* M. tuberculosis* strains, by comparing these to the wild-type parent strain, using the total metabolome extraction method and GC-MS analyses. One of the major findings of their study was the absence of the two saturated mid-chain methyl-branched fatty acids required for mycolic acid synthesis [49]—10Me-C15:0 and 10Me-C16:0—in both of these rifampicin-resistant mutants. This result was accompanied by elevated levels of the well-known* Mycobacterium* branched-chain fatty acid, tuberculosteric acid (TBSA). Considering the mechanism by which these branched-chain fatty acids are synthesized, du Preez and Loots [37] suggested that the* rpoB* mutation results in a disturbance in the equilibrium of mRNA and the ribonucleoside 5′-triphosphates (ATP, GTP, CTP, and UTP), leading to reduced flavin adenine dinucleotide (FAD) required for the synthesis of these essential fatty acids [37, 102]. They furthermore indicated that as a result of the diminishing nucleoside triphosphates (NTPs), ATP in particular, less SAM is synthesized, further contributing to a reduction in these 10-methyl branched-chain fatty acids observed in the mutant strains [37]. These findings were further confirmed by the accumulation of their upstream fatty acid substrates, oleic acid (C18:1 *ω*9C), and palmitoleic acid (C16:1 *ω*7C). The consequential reduction in mycolic acids would be expected to result in a decreased use of lipoarabinomannan (LAM) for cell wall synthesis, resulting in accumulating LAM levels in circulation [103], which explains why the major fatty acid components of LAM, namely, TBSA and C16:0, were detected in elevated concentrations [37]. However, although this hypothesis is a possibility, the accumulation of LAM substrates may also indicate inhibition of LAM synthesis. Lastly, heptadecanoic acid (C17:0) was detected in reduced amounts in the two mutant strains when compared with the* M. tuberculosis* wild-type parent strain. du Preez and Loots [37] ascribed this to the ability of the mutant strains to use acetate and fatty acids as primary carbon sources via the glyoxylate cycle during conditions of stress [104, 105]. The catabolism that was a consequence, resulted in elevated concentrations of propionyl-CoA and acetyl-CoA [106], and in this metabolomics study benzene propionic acid (a derivative of propionic acid) was detected in raised concentrations in the metabolite profile of one of the rifampicin-resistant mutants. This observation confirmed their hypothesis and illustrated the potential of metabolomics and systems biology and its application to understand drug resistance better.

Loots [107] subsequently used the same metabolomics research approach to identify potentially new metabolic pathways and metabolite markers to explain many of the phenotypical characteristics associated with a* katG* mutation and the resulting isoniazid-resistance in* M. tuberculosis*. The isoniazid-resistant strains demonstrated increased susceptibility to oxidative stress and consequently adapted to this by upregulating the synthesis of a number of compounds. These involved the increased uptake and use of alkanes and fatty acids as sources of carbon and energy as well as the synthesis of various compounds and processes directly associated with reducing oxidative stress, including an ascorbic acid degradation pathway. Such a pathway in these organisms has not been proposed before.

## 3. Further Applications of Metabolomics for Improved Characterization of* M. tuberculosis*

The genome of* M. tuberculosis* is constituted of genes that code for the proteins involved in both aerobic and anaerobic respiration [32]. As these tubercle bacilli are predominantly located in the lungs of the host, their survival depends on successfully competing with the host for oxygen [33]. This may be limited during the latent phase of infection, when the microbe is contained within the granuloma of the host's lung. Aerobic respiration by the bacterium is achieved by conventional oxidative phosphorylation eventually to yield ATP via the electron transport chain, involving a ubiquinone cytochrome *b* reductase complex and cytochrome *c* oxidase [33, 105]. Additionally, the necessary components linked to the anaerobic growth of* M. tuberculosis* have also been identified in its genome; when oxygen is depleted, nitrate serves as the terminal electron acceptor. During anaerobic conditions, upregulation in a number of genes has been shown, which include (1)* narX,* which encodes for nitrate reductase; (2)* nark2*, necessary for the transport of nitrite/nitrate; and (3)* fdxA,* which encodes feredoxin A, a protein crucial for alternative electron transport [108, 109]. Furthermore, anaerobic conditions also result in increased activity of ribonucleotide reductase class II (*nrdZ*) [108] as well as nitrite reductase (*nirBD*) [110]. The last uses a rearranged form of the* narGHJI* operon, which is also the operon for nitrate reductase [111]. Although not yet explored in any* Mycobacterium* species, Hirai et al. [112] used a combined postgenetic approach to investigate the gene-to-metabolite networks regulating sulfur and nitrogen as well as the secondary metabolism of the flowering plant* Arabidopsis thaliana* grown in nutritionally stressed conditions. This study illustrated the downregulation of the nitrate reductase genes, following the reduction of anaerobic photosynthetic activity, thereby illustrating the role of these genes during anaerobic conditions [112]. Various plants respond to changing nitrate or nitrite reductase activity, elevated CO_2_ and decreased aerobic photosynthesis rates, by increasing intracellular concentrations of specific metabolites [113], which can be detected by qualitatively and quantitatively comparing the corresponding metabolomes. This study approach could potentially be applied also to investigate these mechanisms in mycobacterial respiration in* M. tuberculosis*.

## 4. Concluding Remarks

Improved understanding of the mechanisms involved in* M. tuberculosis *disease, using a combined systems biology research approach, should reveal new insights into the improved diagnosis and treatment of this devastating epidemic. Different research approaches—genomics, proteomics, and transcriptomics—have successfully contributed to expanding our knowledge and appreciation of these disease mechanisms. Despite the progress made, however, most of the functions of the genes involved are still unconfirmed, partly due to a lack of proteomics and metabolomics data. Over the last decade, systems biology has contributed greatly towards elucidating the underlying mechanisms associated with various infectious diseases [114, 115]. The eventual aim of such a multifaceted approach with regard to the largely uncontrolled TB epidemic is a better understanding of TB disease pathogenesis through the identification of specific genes, related proteins (or enzymes), and metabolite markers. This understanding could potentially be used in the development of improved diagnostic and treatment strategies. Metabolomics combined with functional genomics and proteomics would serve well as a new approach for revealing the mysteries of* M. tuberculosis *and attaining themillennium development goals, as set by the WHO, expanding on the research contributions made so far and summarized in this review.
